# Impact of the COVID-19 pandemic on UK medical school widening access schemes: disruption, support and a virtual student led initiative

**DOI:** 10.1186/s12909-021-02770-0

**Published:** 2021-06-15

**Authors:** Emily R Bligh, Ellie Courtney, Rebecca Stirling, Asveny Rajananthanan, Sheffield Neuroscience Society, Hibatallah Altaher, Joshua Thomas, Rebecca Anim-Boadu, Doha Basiouni, Julian Kurz, Mara-Luciana Floare, Immanuelle O Nyamali, Young Chan, Kawthar A Hussein, Andrew Whitfield, Helia Ghahremani Nezhad

**Affiliations:** Faculty of Medicine, Dentistry & Health, University of Sheffield Medical School, S10 2RX Sheffield, United Kingdom

**Keywords:** Widening access, Medical Education, COVID-19, Medical Students

## Abstract

**Background:**

COVID-19 has disrupted medical education in the United Kingdom (UK). The pandemic may result in a long-term disproportionate negative impact to students applying to Medical School from a low-socioeconomic background. In addition, the upsurge in Medical School applications increases the likelihood of stricter University entry criteria over the coming years. There is no current research to determine how widening participation of Medicine to students from low-socioeconomic backgrounds can be improved virtually. The aim of this study is to establish the impact of COVID-19 on students enrolled in UK widening access schemes and the role of virtual student led initiatives in widening participation.

**Methods:**

A voluntary online survey was distributed to UK Sixth Form students (*N* = 31) enrolled in a widening access scheme who attended Sheffield Neuroscience Society International Virtual Conference in February 2021. The event was free to attend. The five-domain survey consisted of questions determining demographics, career aspirations, impact of COVID-19, academic skillsets and an educational manipulation check.

**Results:**

There were 30 pre-conference and 26 post-conference responses. 76.7 % had work experience cancelled due to COVID-19. A total of 36.7 % of participants reported participating in virtual work experience. ‘Observe GP’ and ‘Medic Mentor’ were each specified as attended virtual opportunities in 20 % of answers. Post conference, students felt significantly more confident in applying to Medical School (*p* = 0.008) and more prepared to undertake a presentation (*p* = 0.002). Educational manipulation check scores increased significantly (*p* = 0.003). 100 % of students felt inspired to do further CV building activities.

**Conclusions:**

COVID-19 has negatively impacted pupils enrolled in UK Medical School widening access schemes. Virtual student led initiatives can instill confidence in delegates from low socio-economic backgrounds, increase their career knowledge and inspire them to take part in further CV building exercises. Both Medical Schools and medical students play a key role in widening participation. This study recommends Medical Schools promote access to virtual events, urge private and state schools to declare offered opportunities and act mindfully when determining student’s academic potential in the context of their socioeconomic and/or educational background.

**Supplementary Information:**

The online version contains supplementary material available at 10.1186/s12909-021-02770-0.

## Introduction

COVID-19 has caused an unprecedented disruption to medical education in the United Kingdom (UK)[1]. Prior to the COVID-19 pandemic, students from lower socioeconomic backgrounds experienced greater challenges when applying to Medical School[2]. Unfortunately, in light of the COVID-19 restrictions, the introduction of full-time virtual learning has further exacerbated the gaps in opportunity between different societal groups[3]. Furthermore, this educational transition adds pressure to families who face the challenge of balancing financial and homeschooling difficulties[4]. The aforementioned learning environment is likely to have long-term impacts for disadvantaged school children and university students in years to come.

Ensuring Universities train doctors from a variety of backgrounds is pivotal in ensuring doctors represent the communities for which they provide healthcare[5]. Medical students predominantly originate from a high socio-economic background which alludes to the longstanding issue that those from lower socio-economic classes are underrepresented in Medicine[3, 6]. In 2019, the Medical School’s Council (MSC) report recognised that certain demographic characteristics remain small and there is still progress to be made. These underrepresented groups include those coming from neighbourhoods of low participation in higher education, receiving free school meals/income support or not having university-educated parent[7].

In an effort to combat bias and accommodate for these disadvantages, medical schools often give contextualised offers to students[8]. The MSC released a statement in February 2021 following a sharp increase in applications and consequently stricter selection criteria declaring, ‘Medical schools will continue to consider contextual information when making admissions decisions to ensure that applicants from underrepresented backgrounds are not further disadvantaged by the upsurge in applications’[9]. This statement warrants further investigation and thus knowledge is required to examine exactly how much COVID-19 is likely to impact a student’s academic potential based upon their socioeconomic and/or educational background.

Widening access schemes (WAS) are defined as programmes that aim to raise awareness, aspirations and achievement levels of eligible students by offering support and guidance through a range of medicine related activities[10]. Medical schools are obligated to provide an outreach scheme[11], to address issues that may prevent students from applying to medicine. These programmes can include mentoring programmes, workshops and residential summer schools. Student led face-to-face initiatives have also previously been used as an effort to widen participation[12]. The disruption caused by COVID-19 in both medical school and medical student efforts to widen participation accompanied by current funding cuts negate further research to attain the current circumstances and level of support provided[13].

There are currently no research studies determining the effects of COVID-19 on widening participation students nor has there been any research to determine the impact of virtual student led initiatives in approaching this issue. Therefore, this paper aims to address both of these issues. Firstly, this research sets out to determine the impact of COVID-19 on opportunities for WAS students. Secondly, the aim is to establish the short-term impact of a virtual student led initiative on WAS students.

## Methods

### Design

An online pre- and post-conference survey consisting of 11 questions was electronically distributed to Sixth Form pupils (*N* = 31) enrolled in the University of Sheffield Discover Medicine WAS at the February 2021, Sheffield Neuroscience Society International Virtual Conference (see Additional file 1). The five-domain survey consisted of questions determining demographics, career aspirations, impact of COVID-19, academic skillsets (preparedness to undertake a presentation) and an educational manipulation check. Importantly, delegates were asked directly how confident they were in applying to medical school. The survey consisted of Likert 10-point closed ended questions, multiple choice questions and open short answer questions. The educational manipulation check of lecture content was based upon knowledge of careers, neuroscience and presentation skills which is identified as Q13-16 in the pre-conference survey and Q11-14 in the post-conference survey. A final question determined the opportunity for follow up, as such, application status of these students can be followed prospectively. Responses were matched with a unique code. The survey was prepared by authors and modified following the pilot survey on widening access students at the Sheffield Neuroscience Society 2020 conference. Data was stored and analysed in Google Forms, Microsoft Excel and SPSS (IBM Version 26). Significance testing was performed using Wilcoxon Matched Pairs Tests and Paired-Samples T-Test with statistical significance set at *P* < 0.05.

### Setting

Sheffield Neuroscience Society is a student led sub-society of the University of Sheffield Medical Society. The virtual conference was free to attend and the schedule included key note lectures and workshops dedicated to neurosurgery and neurology as depicted in Table 1. Advertisement was achieved through social media; posts were subsequently shared by undergraduate neuroscience societies across the UK.

**Table 1:** Sheffield Neuroscience Society one-day conference structure

Session	Topic
Key Note Lectures	1.Neurology: Cognospeak™: An Automated Assessment for Patients with Memory Problems 2. Neurosurgical Oncology: Current Concepts and Future Challenges 3. Intensive Neurorehabilitation augmented by Robotics and Virtual Reality 4. Leveraging Brain Rhythms as a Therapeutic Intervention for Alzheimer’s disease
Opportunity to Present	Poster/Oral
Workshops	1. Pathway to Neurosurgery 2. Presentation Skills 3. Pathway to Neurology

### Conference Structure

#### Participants

The University of Sheffield Discover Medicine team directly contacted students, who were eligible for widening participation, with details of the opportunity and the local widening participation society shared the event on their Facebook page. Eligibility criteria meant that all participants in the study were from a state school in South Yorkshire (and the surrounding regions) and are the first generation of their family to enter Higher Education (HE), (other than siblings, or parents who attended HE as a mature student, aged over 21). The voluntary survey was emailed to students who identified themselves as a widening access pupil on the sign-up form.

#### Ethics

 Ethical approval was attained through the University of Sheffield Ethics Committee. A participant information sheet including a General Data Protection Regulation (GDPR) statement was included in the questionnaire (see Additional file 1) and all responses were made anonymous. Students were informed that participation in the survey would not effect their chances of medical school entry.

## Results

### Delegate Demographics

30 Responses were received to the pre-conference survey and 26 responses to the post-conference survey from pupils enrolled in the Discover Medicine Widening Access Scheme. All students indicated they were in Year 12 (aged 16–17), the penultimate year of UK education prior to University. 90 % of participants were eligible for the widening participation scheme because they ’Are from a low progression to Higher Education area and the lowest socio-economic groups’ whilst the remaining 10 % ‘have individual circumstances that mean they will need to overcome other barriers to learning/progression’. 26.7 % of students had previously attended a student led medical conference.

### Impact of COVID-19

**Fig. 1:**
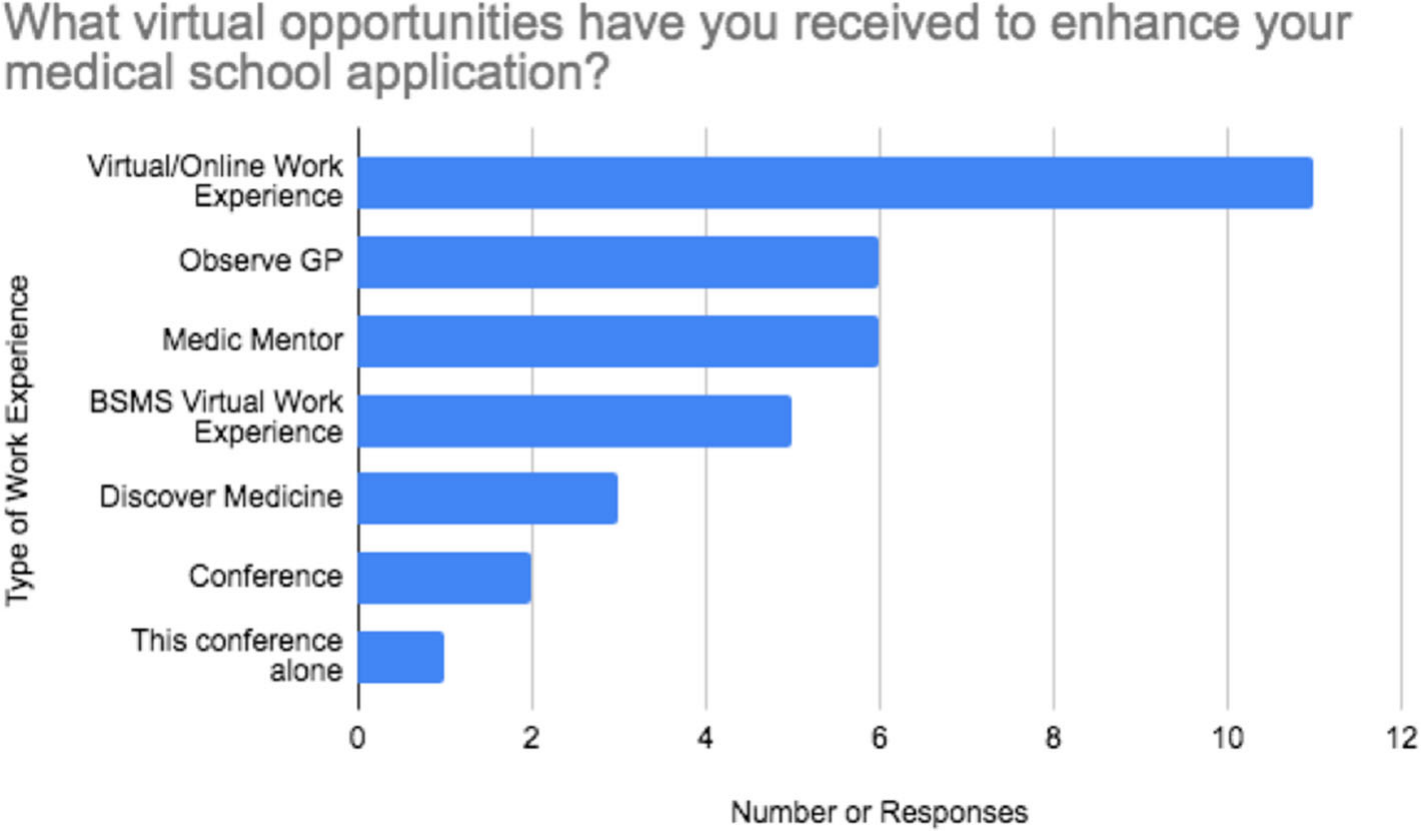
A bar chart demonstrating participant short answer responses when asked ‘What virtual opportunities have you received to enhance your medical school application?’. BSMS = Brighton and Sussex Medical School.

Twenty-three delegates (76.7 %) had work experience cancelled due to COVID-19. When asked ‘What virtual opportunities have you received to enhance your medical school application?’, 36.7 % of students responded with virtual/online work experience. ‘Observe GP’ and ‘Medic Mentor’ were each specified in 20 % of answers. One student declared this was their only virtual opportunity they have participated in. Further types of opportunities are recorded in Fig. 1.

### Career Aspirations

**Fig. 2:**
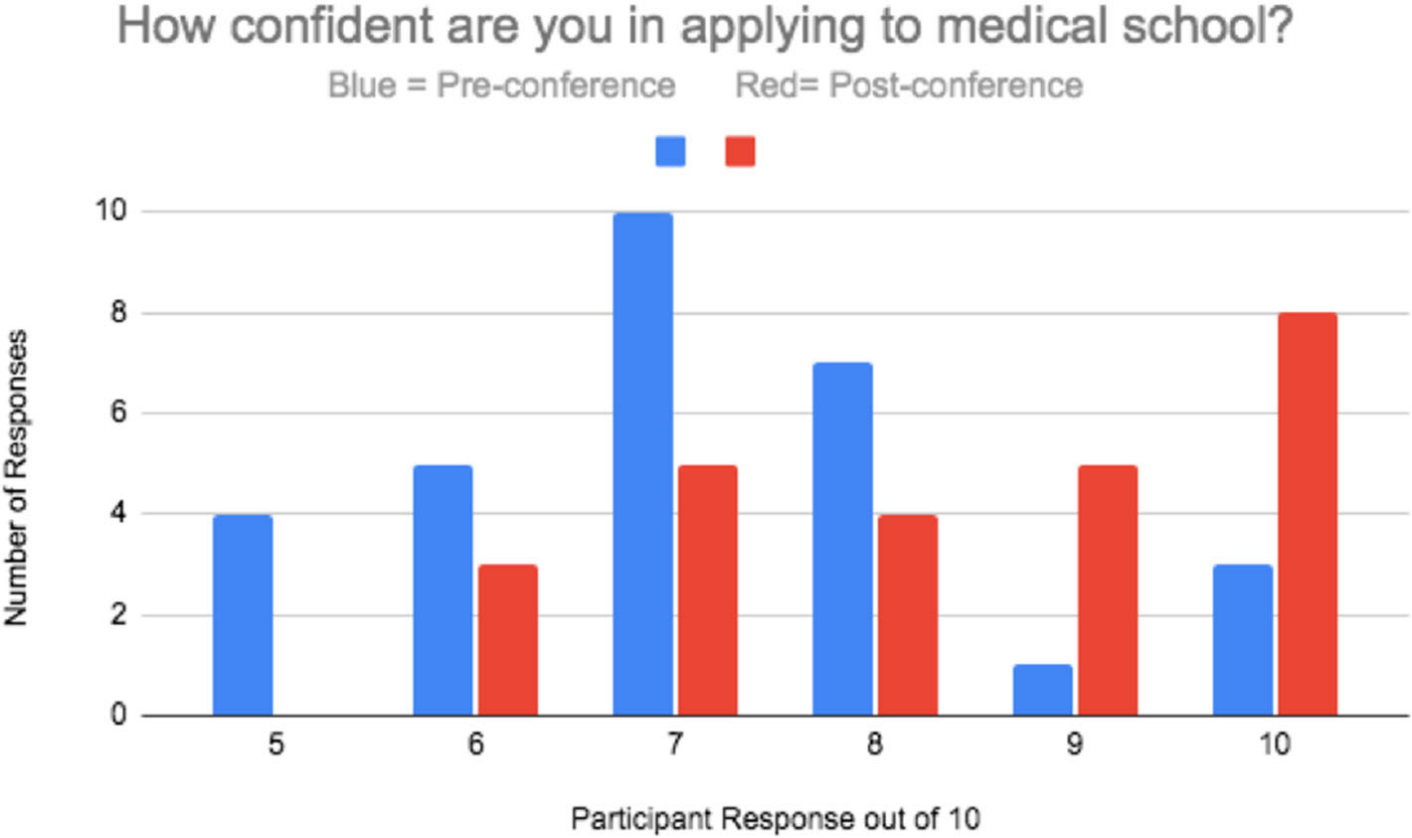
A bar chart depicting participant response to the question ‘How confident are you in applying to medical school? 0–10’.

When asked ‘How confident are you in applying to medical school?’ matched responses increased significantly (p = 0.008) from a median of 7/10 (mean = 7.2, range = 5–10) to 9/10 (mean = 8.4, range = 6–10), as shown in Fig. 2. The median pre-conference likeliness of applying to medical school was 10/10 (mean = 9.6, range = 7–10). Post-conference, this remained 10/10 (mean = 9.8, range = 8–10). A total of 63.3 % of WAS students had originally offered the maximum score for this question therefore only small increases in range and mean average were seen. Interest in a neuroscience career was a pre-conference median of 8/10 (mean = 7.7, range = 5–10) and a post conference median of 8/10 (mean = 8.2, range = 5–10), this change was not significant (*p* = 0.219). Those who had not originally indicated the maximum score pre-conference were significantly more interested in a neuroscience career post-conference (*P* = 0.05).

100 % of participants believed this conference inspired them to do further online CV building exercises.

### Academic Skillsets

Preparedness to undertake a presentation in matched responses increased significantly (p = 0.002) from a median of 6/10 (mean = 5.7, range = 1–9) to 8/10 (mean = 7.6, range = 4–10). Matched educational manipulation check scores (*N* = 15) increased significantly (*p* = 0.003) from a mean average of 1.8/4 to 3.2/4.

## Discussion

### Implications

COVID-19 has resulted in cancellation of work experience for WAS students. Work and/or voluntary experience is critical to the success of hopeful candidates, allowing them to reflect on their experiences in interview and show dedication to the profession[14, 15]. However, during the pandemic most face-to-face opportunities have halted[16]. Nevertheless, given that medical careers often run in the family, students with a Parent as a Doctor may still have access to opportunities through hospital connections[17]. Fortunately, the Medical Schools Council has provided online guidance on how to gain relevant experience during the pandemic, although the reach of this document, with any virtual information, is yet to be determined[16].

Positively, innovations such as Virtual Work experience, ‘Observe GP’ and ‘Medic Mentor’ have acted to support students enrolled in widening participation schemes. The above-mentioned organisations such as the ‘Medic Mentor’ have developed free live virtual work experience to hopeful medical students in the UK. Although a great initiative, places are limited and rely on an application process[17]. In many disadvantaged regions, schools lack a nominated careers adviser specifically for medicine, contrasting to the majority of fee paying independent schools[4]. Consequently, disadvantaged pupils may have a less refined application statement, further limiting their ability to take part in the virtual platforms mentioned previously.

Although many participants reported attending other virtual opportunities, some students were not as fortunate. One obstacle low-income students will encounter includes access to a reliable and stable internet connection, thus preventing students accessing relevant learning materials[4]. This exasperates the previously established disparity in educational attainment amongst social groups in the UK[18]. Additionally, teenagers from disadvantaged backgrounds may not have access to the technological devices and or the appropriate quiet learning environment[19]. Moreover, with parents working from home the only available family device may have to be used to maintain an income for the household rather than educational purposes which, in the worsening economic climate, takes priority[4]. Ultimately, the aforementioned factors impact students ability to maintain and achieve their academic attainment which is a crucial requirement for admission into medical school. The requirement for pupils to reach their target grades, typically A (the second highest achievable grade) in 3 subjects, is paramount given the conflicting evidence on how pupils will be awarded grades fairly using the government’s algorithms[20].

### Recommendations for Medical Students

The virtual student led initiative studied in this article proved valuable by instilling confidence in student’s medical school application, increasing perceived preparedness to undertake a presentation and inspiring participants to do further CV building activities. Furthermore, students built knowledge relating to careers, neuroscience and presenting. In lieu of our findings we recommend medical student led societies incorporate WAS inclusive events into their annual schedule where possible. Enabling access to student led initiatives free of charge for widening participation students allows attendees the chance to explore their interests further but also provides them with evidence of their insight into the profession in the form of a certificate for application purposes.

The chief limitation of this virtual student led initiative is that delegates required a stable internet connection to attend the conference. Students without internet or with one shared household device are likely to be in the lowest-socio economic groups and are therefore less likely to be able to attend[21]. We suggest recording virtual student led initiatives to enable WAS students with limited access to the required technology more equal opportunity. This would allow the student to watch the event at a time appropriate for their household, for example after a parent has finished working from home on the only family device. Furthermore, as the event would not have to be watched live, any problems caused by a poor internet connection would not result in the student missing the event. However, this limits the student’s engagement as they would not be able to ask any questions to the speakers[22]. Therefore, it is also important to maintain face-to-face opportunities where possible, as to not stretch inequality gaps between WAS students.

Finally, we recommend current medical students reach out to low performing schools and offer their support to aspiring pupils in the form of online support. Participation in mentoring schemes is associated with a higher medical school offer rate[23]. Offering personal statement advice or conducting a mock interview could instil confidence in the students and give them access to the opportunities that are readily available at independent schools[24]. Giving an insight into university life may also motivate and raise the aspirations of applicants who come from a background lacking higher education culture[8]. Furthermore, the experience of teaching and communication may prove extremely valuable to the medical student involved[25].

In total, it is suggested medical students harness their altruism and where budget and time constraints allow, support WAS schemes by mentoring and/or incorporating WAS students into free-of-charge undergraduate events.

### Recommendations for Medical Schools

A large proportion (78.6 %) of Year 12 students from this study faced cancellation of face-to-face work experience. Medical schools profoundly influence widening participation to Medicine, as such, Universities should be particularly mindful of the negative impact of COVID-19 on WAS students and offer special consideration to these students[7]. Students without a higher educated parent are likely to have a lower level of educational support during the school closure period[4]. Moreover, these parents are less likely to work as healthcare professionals meaning attaining work experience should their child have an opportunity cancelled, could be more difficult[14]. Thus, Medical Schools should take this into account whilst carefully reviewing applicant grades and personal statements. One approach could be assessing applications more holistically and offer increased weighting to the aptitude tests, such as the University Clinical Aptitude Test (UCAT), which are less influenced by socioeconomic factors and barriers to virtual learning[26].

A further consideration for Medical Schools, is how the above-mentioned issues may impact admission interviews for Medical School. The economical savings of a virtual interview may not outweigh the implications, with various potential technical issues affecting the candidate’s performance[27]. In addition, virtual interviews may expose disadvantaged pupils to unconscious bias from interviewers, following exposure to the home environment[28]. Currently interviewees at certain Medical Schools are given the questions prior to interview, in order to prevent bias of preparation courses at independent schools[29]. The technical issues and the exposure to the home environment, could retract previous efforts.

Furthermore, given the evident influx in virtual opportunities offered to WAS students and the positive outcomes of the virtual initiative in this study, as such, Medical Schools should enable access of such events to ensure those without a stable internet connection/access to an appropriate device are not disadvantaged. For example, by offering recorded talks, University Library day passes for computer access or the option of a University laptop loan. Although virtual opportunities play a role in supporting widening access schemes, engaging in face-to-face opportunities should be maintained where possible to ensure student’s exposure to Multi-Disciplinary Team (MDT) working in a hospital/community healthcare environment[30].

Importantly, we recommend medical schools ask state and private school students to declare the online opportunities they have offered their students. Given that independent school students have been provided with more online lessons, online platforms and spend per student, it is imperative institutions are transparent[31]. In aim to combat bias and privilege, this transparency will allow medical schools to more equally compare candidates attained career building activity and grades.

In summary, recommendations for Medical schools include continuing to encourage widening access students through schemes consisting of; engaging face-to-face activities where possible, accessible virtual events and special consideration of A-level grades and attained work experience. Furthermore, we request transparency from school’s by clearly presenting offered opportunity, allowing for fairer assessment on an individual basis.

### Limitations

Although highly relevant, nonetheless, this small-scale study is limited by it’s single region, subjective nature and power in outcome data. Furthermore, there is no data to compare pupils enrolled in widening access schemes to those who are not. To determine objective impact, students will be followed up at 6–12 months to establish their application status to Medical school as part of the prospective study. Involvement of multiple centers is planned given the positive outcome of this initial study.

## Conclusions

COVID-19 has resulted in cancellation of work experience opportunities for Year 12 students enrolled in Widening Access Schemes. The effects of the pandemic are likely to disproportionately effect pupils from a low socio-economic background. Virtual student led initiatives can instill confidence in delegates from such backgrounds and build their career knowledge, as well as inspiring them to do further CV building exercises. Medical Schools and medical students both play an important role in strengthening widening participation schemes. This study recommends these parties dedicate time to reviewing and improving current virtual and face-to-face opportunities. Strategies should be initiated to promote access to virtual events for those without internet or access to an appropriate technological device. Medical Schools should be particularly mindful of the effects of COVID-19 when assessing student’s academic potential in the context of their socioeconomic and/or educational background. To combat bias and privilege, they should urge state and private schools to declare online opportunities offered to their students. The proposed prospective study is likely to better determine the long-term effects of virtual student led initiatives.

## Supplementary Information

**Additional file 1:** Pre-conference Questionnaire. Post-conference Questionnaire

## Data Availability

The datasets generated and/or analysed during the current study are not publicly available due to the sensitive nature of financial information from high risk participants (Under 18yrs). However, data is available from the corresponding author on reasonable request.
