# Supplementary material for: Impact of the COVID-19 pandemic on UK medical school widening access schemes: disruption, support and a virtual student led initiative
Source: BMC Med Educ. 2021 Jun 15;21:344. doi: 10.1186/s12909-021-02770-0 (PMC8203309; doi:10.1186/s12909-021-02770-0)
Supplement: Supplementary file 1 — Additional file 1: Pre-conference Questionnaire. Post-conference Questionnaire [file 12909_2021_2770_MOESM1_ESM.zip › Discover Medicine Post Conference Questionnaire - Google Forms.pdf]

Discover Medicine Post-Conference Survey

The following survey is led by Sheffield NeuroSoc. All responses will be made anonymous. The aim of this voluntary questionnaire is to understand the role student led conferences play in determining widening participation to medicine and the impact of COVID-19 on widening access scheme enrolled students. We will be surveying participants before and after the conference to create a comparison of results, as well following up students with a further survey in 6-24 months should you consent. . Please answer the questions honestly. We aim to publish the results in the near future and data will be stored electronically. Queries can be answered by Sheffield Neuroscience Society committee members at the conference or by contacting ebligh1@sheffield.ac.uk. Thank you

Please note: You may withdraw from the study at any point. Completing the survey/or withdrawing will not impact on your chance of getting into Medical School

Will my taking part in this project be kept confidential? All information that is collected about you during the course of the research will be kept strictly confidential. The information you give will
not be used in any way that could identify you and no one outside the research team will know who
took part in the survey. Survey responses will be stored separately from email addresses and only
those researchers that need to send out the next survey will have access to your email address.

Who is the data controller? Sheffield Neuroscience Society are responsible for looking after your information and using it
properly.

What is the legal basis for processing my personal data? According to data protection legislation, we are required to inform you that the legal basis we are applying in order to process your
personal data is that 'processing is necessary for the performance of a task carried out in the public
interest' (Article 6(1)(e)). Further information can be found in the University's Privacy Notice

https://www.sheffield.ac.uk/govern/data-protection/privacy/general.

*Required

1. Email address *

2. How likely are you to apply to medical school? *

Mark only one oval.

1	2	3	4	5	6	7	8	9	10

3. How confident are you in applying to medical school? *

Mark only one oval.

1	2	3	4	5	6	7	8	9	10

4. How interested in a career related to neuroscience are you? *

Mark only one oval.

1	2	3	4	5	6	7	8	9	10

5. How prepared do you feel to undertake a presentation? *

Mark only one oval.

1	2	3	4	5	6	7	8	9	10
◯	◯	◯	◯	◯	◯	◯	◯	◯	◯

6. Has this conference inspired you to do further online CV building exercises? *

Mark only one oval.

◯ Yes

◯ No

7. Rate the conference out of 10

Mark only one oval.

1	2	3	4	5	6	7	8	9	10
◯	◯	◯	◯	◯	◯	◯	◯	◯	◯

8. What went well?

9. What could be improved?

10. Did you fill out the pre-conference 4 question educational manipulation check?

Mark only one oval.

- Yes - Fill out the next 4 questions
- No - Fill in the last question on the survey

11. How many years run through training does neurosurgery take at minimum?

Mark only one oval.

- 3
- 5
- 7
- 8
- 9

12. Neuroplasticity is...

Mark only one oval.

- The texture of the brain following injury
- The corticospinal tract pathway
- the ability of the brain to form and reorganize synaptic connections, especially in response to learning or experience or following injury.
- The ability for the brain to recognise objects by touch without visualising them
- The direction of fibres travelling through cranial nerves

13. Cyclin-dependent kinase 5 (Cdk5) is...

Mark only one oval.

- brain-specific protein serine/threonine kinase essential for brain development, synaptic plasticity, learning, and memory.
- Novel drug used to treat Parkinson's
- An anatomical structure found in the cervical vertebral bodies
- An anti-inflammatory DMARD used in the treatment of metastatic cord compression

14. A P3 (cubed) presentation focusses on...

Mark only one oval.

- the powerpoint (p1), the speech (p2) and the audience (p3)
- the idea (p1), the preparation (p2) and the delivery (p3)
- the presenter (p1), the audience (p2) and the patient (p3)
- the story (p1), the supportive media (p2) and its delivery (p3)

15. What day of the month is your birthday? What are the first three letters of your mother’s first name? This is to ensure we can link responses anonymously through a unique code. E.g 14/09/1995 and Mother's name is Caroline = 14car. *

This content is neither created nor endorsed by Google.

Google Forms
